# Gene expression profile analysis of ileum transcriptomes in pigs fed *Gelsemium elegans* plants

**DOI:** 10.1038/s41598-019-52374-4

**Published:** 2019-10-31

**Authors:** Chong-Yin Huang, Kun Yang, Jun-Jie Cao, Yu-Juan Li, Zi-Yuan Wang, Hui Yu, Zhi-Liang Sun, Xiaofeng Zheng, Zhao-Ying Liu

**Affiliations:** 1 Hunan Engineering Technology Research Center of Veterinary Drugs, Hunan Agricultural University, Changsha, 410128 China; 2 College of Veterinary Medicine, Hunan Agricultural University, Changsha, 410128 China

**Keywords:** Sequencing, Transcriptomics

## Abstract

*Gelsemium elegans* is a flowering plant in the Loganiaceae. Because it can promote the growth of pigs and sheep, it is widely used, including in veterinary clinics, but little information is available about its biological effects. Here, we used high-throughput sequencing to characterize the differentially expressed genes (DEGs) in the ileums of pigs between a control group and a group fed *Gelsemium elegans* for 45 days. We found that *Gelsemium elegans* affected many inflammatory and immune pathways, including biological processes such as defense responses, inflammation and immune responses. Moreover, this study identified several important genes related to the anti-inflammatory activity of *Gelsemium elegans* (e.g., CXCL-8, IL1A, and CSF2), which will be beneficial for further study of the pharmacological mechanisms and clinical applications of *Gelsemium elegans*.

## Introduction

*Gelsemium* is an herb in the family Loganiaceae. *Gelsemium* mainly includes three species with different regional distributions: *Gelsemium sempervirens* (L.), *Gelsemium rankinii* Small, and *Gelsemium elegans* Benth. *Gelsemium elegans* is mainly distributed in southeast Asia and has been used as a traditional Chinese medicine for treating neuropathic pain, rheumatoid pain, spasms, skin ulcers and cancer for many years^1,2^. In China, the plant is mainly distributed near the middle and lower reaches of the Yangtze River, especially in the warm coastal areas of the Fujian and Guangxi provinces. However, *Gelsemium elegans*, a poisonous plant, exerts obvious toxicity toward the human body. Poisoning has often been reported after people have eaten *Gelsemium elegans* by mistake^3^. After poisoning, patients suffer from symptoms such as dizziness, sweating, nausea, vomiting, muscle relaxation, limb paralysis, dyspnea, coma and convulsion^4,5^. The nervous system is depressed, and death is caused by respiratory depression^4–6^; This toxicity limits the applications of this plant.

Interestingly, in Chinese folk medicine, *Gelsemium elegans* is often used in animals to prevent parasite infestation and to promote growth. Its effect is obvious in pigs and sheep. In the Compendium of Materia Medica, it is recorded that people who eat *Gelsemium* by mistake die, while sheep that eat *Gelsemium* grow large and fat^7^. Chen *et al*. recorded that *Gelsemium*, which is regularly given to pigs and sheep, can invigorate the stomach, kill parasites and fatten the animals^8^. These statements show that *Gelsemium* not only is nontoxic when eaten by animals such as pigs and sheep but also has the effects of fattening the animals and preventing disease. Therefore, it is critical to study the mechanisms of the effects of *Gelsemium elegans* on the pig intestinal tract.

In this experiment, high-throughput transcriptome sequencing technology was used to examine changes at the transcriptome level in the pig ileum under the influence of *Gelsemium elegans*. High-throughput sequencing has characteristics including a short sequencing time, low cost, and high sequence quantity^9^. Transcriptomics, an important component of functional genomics research, can be used to study overall gene function and structure, revealing specific biological processes and molecular mechanisms that are involved in the process of disease occurrence^10^. Therefore, the purposes of this study were to clarify the possible mechanisms of the effects of *Gelsemium elegans* on the intestinal tract as a whole based on high-throughput sequencing, to provide new data to reveal the molecular mechanisms of the pharmacological effects of *Gelsemium elegans* and to provide important references for further in-depth study of the mechanisms of the effects of *Gelsemium elegans*.

## Results

### Transcriptome sequencing data quality testing

Transcriptome sequencing was performed on a total of 6 samples from the experimental group and the control group (Table 1), and an average of 52.168 M reads were obtained. After removing the linker fragments and the low-quality fragments, a total of 51.171 M high-quality clean reads were obtained; the Q20% of the clean reads in each sample was above 96.80%, and the Q30% was above 91.60%, indicating that the sequencing quality was good and provided good original data for subsequent data assembly. Upon comparing the clean reads to the reference genome, an average of 49.135 M could be mapped, which accounted for 96% of the total number of sequences. The comparison showed that the alignment efficiency was good.

**Table 1:** Statistics from digital gene expression sequencing.

Sample_name	C1	C2	C3	G1	G2	G3
Raw reads	53351960	52203452	62621280	50860180	53702840	40267710
Clean reads	52354682	50836840	61569634	49898956	52727580	39636068
Clean bases (Gb)	7.39	7.24	8.70	7.05	7.49	5.63
Q20(%)	97.10%	96.95%	97.10%	96.80%	96.95%	97.45%
Q30(%)	92.20%	91.90%	92.20%	91.60%	91.80%	92.95%
Total mapped	50591699 (96.60%)	49185332 (96.80%)	58949697 (95.70%)	47391403 (95%)	50409079 (95.60%)	38280432 (96.60%)
Multiple mapped	2682859	2066081	3841841	3278765	3483805	2173146
Uniquely mapped	47908840	47119251	55107856	44112638	46925274	36107286
Reads map to ‘+‘	23954389	23559590	27553907	22056319	23462604	18053653
Reads map to ‘-‘	23954451	23559661	27553949	22056319	23462670	18053633
Non-Splice reads	26179110	26211625	31002946	24250115	26227333	20306368
Splice reads	21729730	20907626	24104910	19862523	20697941	15800918

C1~3: ileum tissue samples from the control group; G1~3: ileum tissue samples from the experimental group.

### Gene expression statistics

HTSeq^11^ was used to quantify the expression level of each sample gene to obtain the raw counts, which were the input data for the next differential expression analysis. Then, the raw counts for each sample were standardized to obtain the FPKM value of each sample gene, and the expression levels of six sample genes were determined (Table 2). The number of highly expressed genes (FPKM > 50) in each ileum sample was maintained at approximately 800, while the number of genes with low expression or no expression (FPKM < 1) was above 7000 in all samples.

**Table 2:** The number of genes in different FPKM expression intervals in all samples.

Group	FPKM interval					
	0~1	1~5	5~10	10~20	20~50	>50
C1	7510 (37.81%)	4396 (22.13%)	2955 (14.88%)	2350 (11.83%)	1690 (8.51%)	961 (4.84%)
C2	7754 (39.04%)	4578 (23.05%)	2760 (13.9%)	2268 (11.42%)	1525 (7.68%)	977 (4.92%)
C3	8249 (41.53%)	4632 (23.32%)	2622 (13.2%)	2040 (10.27%)	1403 (7.07%)	916 (4.61%)
G1	8087 (40.72%)	4722 (23.77%)	2785 (14.02%)	2066 (10.4%)	1360 (6.85%)	842 (4.24%)
G2	8035 (40.45%)	4641 (23.37%)	2718 (13.68%)	2156 (10.85%)	1416 (7.12%)	896 (4.51%)
G3	7602 (38.27%)	4620 (23.26%)	2905 (14.63%)	2380 (11.98%)	1467 (7.39%)	888 (4.47%)

### Screening of differentially expressed genes

Taking a |log2 (fold change)| ≥ 1, a P-value ≤ 0.05 and an FDR (adjusted for P-value) ≤ 0.05 as the screening conditions, the experimental group and the control group were compared and analyzed. A total of 446 differentially expressed genes (DEGs) were screened in this experiment, of which 237 genes were upregulated and 209 genes were downregulated.

### GO classification of differentially expressed genes

**Figure 1:**
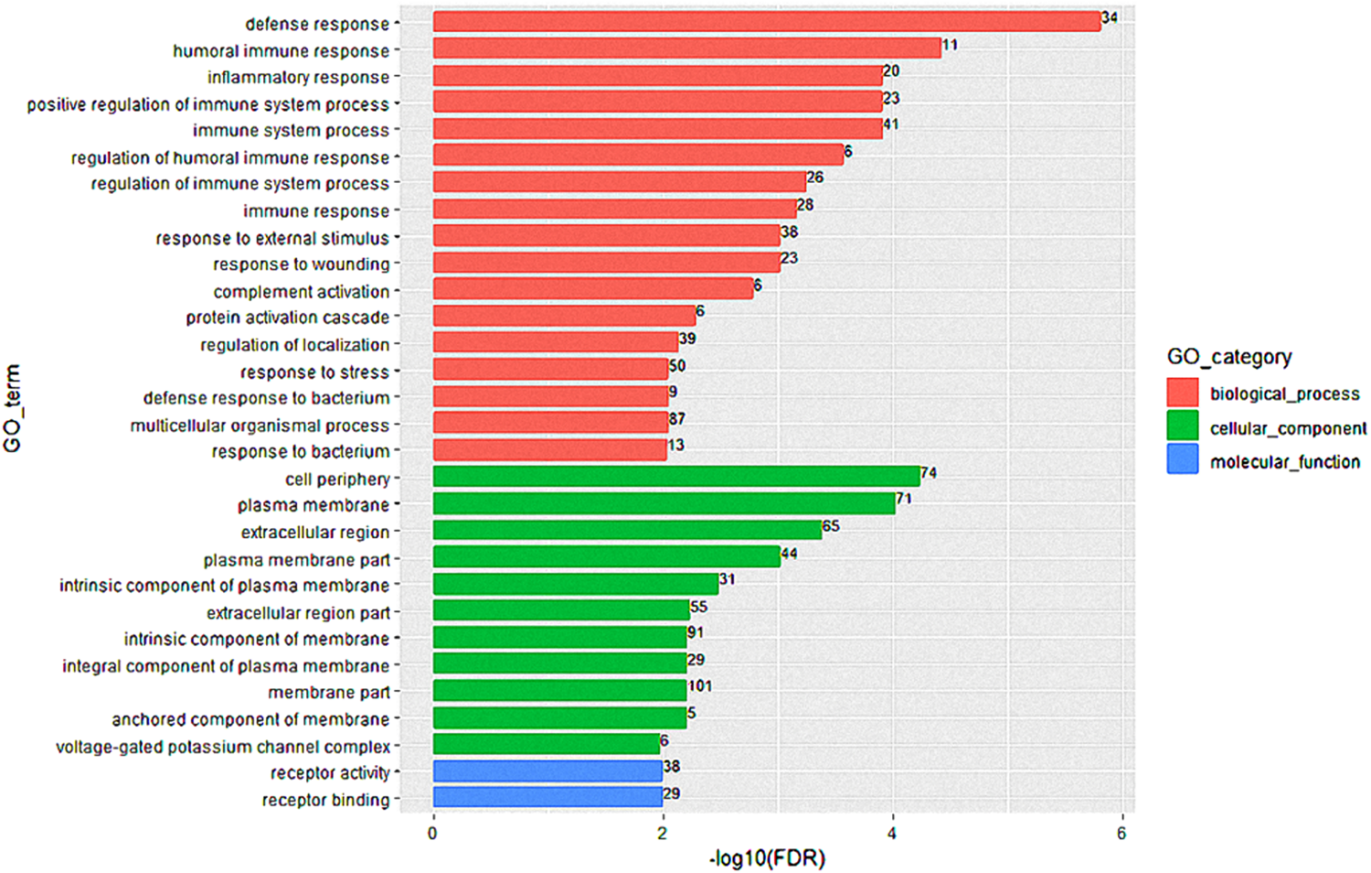
GO classifications of the differentially expressed genes in intestinal tissues from the *Gelsemium elegans* group and the control group. The differentially expressed genes were classified into three categories: biological process, cellular component, and molecular function.

GO annotation was conducted for the DEGs, and a total of 30 significant GO terms were enriched. As shown in Fig. 1, the DEGs were mainly enriched in biological processes such as defense responses, humoral immune responses, inflammatory responses, and immune system processes, which indicates that intake of *Gelsemium elegans* leads to changes in immune and inflammatory response processes in animal bodies. In the cellular component category, the cell periphery, plasma membrane, extracellular region and plasma membrane part terms were most prominent; in the molecular function category, receptor activity/receptor binding was the main term.

### Enrichment of differentially expressed gene pathways

**Figure 2:**
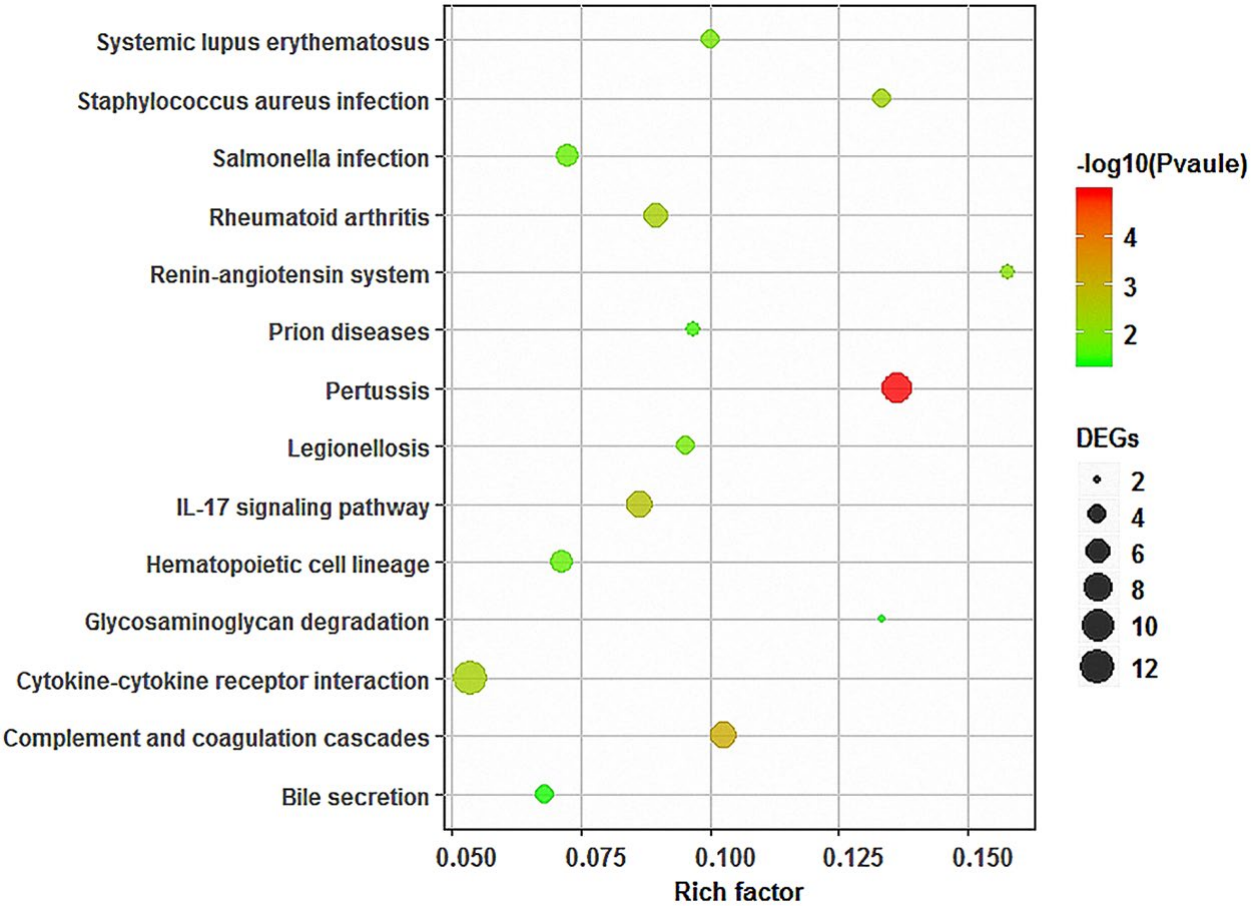
Pathway enrichment results of the differentially expressed genes. The X-axis represents the enrichment factor value, and the Y-axis represents the pathway name. The color represents the P-value (significance: P ≤ 0.05), and the size of the point represents the number of genes. The enrichment factor represents the ratio of the enriched genes to the background genes.

As shown in Fig. 2, 446 DEGs between the experimental group and the control group were enriched in signaling pathways related to inflammation and the immune response, including the complement and coagulation cascade pathway, the IL-17 signaling pathway, the rheumatoid arthritis pathway and the hematopoietic cell lineage pathway, with relatively high enrichment rates.

The genes enriched in the pathways are shown in Table 3. Statistical analyses showed that CXCL-8, IL1A, C3, C5, CSF2, C2, CXCL-2, FOS, AMCF-II, and C8G were involved in at least three of the above pathways, suggesting that these genes may be important hubs for communication between different signaling pathways. Further statistical analyses showed that CXCL-8, CSF2 and AMCF-II are all involved in the cytokine–cytokine receptor interaction pathway and the rheumatoid arthritis pathway, which are related to inflammation, suggesting that these two pathways may be key pathways in inflammation-related reaction processes.

**Table 3:** Pathway enrichment gene expression.

Gene symbol	Gene description	Gene ID	Gene family	Log_2_(Fold Change)	P value
IL1A	interleukin 1 alpha	ENSSSCG00000008090	IL-1 family	−1.12	0.0063
IL1RL2	interleukin 1 receptor like 2	ENSSSCG00000023550	—	3.55	0.0002
IL20RA	interleukin 20 receptor subunit alpha	ENSSSCG00000004157	chemokine receptor family	−1.16	0.0146
IL5RA	interleukin 5 receptor subunit alpha	ENSSSCG00000011528	chemokine receptor family	−1.36	0.0191
CCL28	C-C motif chemokine ligand 28	ENSSSCG00000016871	Intercrine beta(chemokine CC)family	−1.37	<0.0001
CCR2	C-C chemokine receptor type 2	ENSSSCG00000024311	—	−1.16	<0.0001
TNFRSF11A	TNF receptor superfamily member 11a	ENSSSCG00000004898	tumor necrosis factor receptor superfamily	−1.15	<0.0001
LTA	Sus scrofa lymphotoxin alpha (LTA), mRNA.	ENSSSCG00000001403	tumor necrosis factor family.	2.61	0.0289
CSF2	granulocyte-macrophage colony-stimulating factor precursor	ENSSSCG00000023737	GM-CSF family	−2.02	0.0003
FOS	proto-oncogene c-Fos	ENSSSCG00000002383	FOS subfamily	1.06	<0.0001
CXCL8	Sus scrofa C-X-C motif chemokine ligand 8 (CXCL8), mRNA.	ENSSSCG00000008953	CXC chemokine family	−1.21	0.0007
CXCL2	C-X-C motif chemokine 2 precursor	ENSSSCG00000008959	CXC chemokine family	−1.73	0.0011
AMCF-II	Alveolar macrophage chemotactic factor 2	ENSSSCG00000008957	CXC chemokine family	−2.17	<0.0001
INHBA	Inhibin beta A chain	ENSSSCG00000035077	TGF-β family	−1.09	0.0083
DEFB1	Sus scrofa defensin beta 1 (DEFB1), mRNA.	ENSSSCG00000029990	Beta-defensin family	1.67	0.0051
S100A8	Sus scrofa S100 calcium binding protein A8 (S100A8), mRNA.	ENSSSCG00000006590	S-100 family	−3.05	0.0074
ANPEP	alanyl aminopeptidase, membrane	ENSSSCG00000001849	peptidase M1 family	1.01	0.0113
CD38	ADP-ribosyl cyclase/cyclic ADP-ribose hydrolase 1	ENSSSCG00000008742	ADP-ribosyl cyclase family	−1.03	<0.0001
C4BPB	complement component 4 binding protein beta	ENSSSCG00000015661	—	−1.64	<0.0001
C4BPA	Sus scrofa complement component 4 binding protein, alpha (C4BPA), mRNA.	ENSSSCG00000015662	—	−1.18	0.0110
C8G	complement C8 gamma chain	ENSSSCG00000005840	calycin superfamily	2.17	0.0217
F11	—	ENSSSCG00000032653	peptidase S1 family	−1.71	0.0241
C2	complement C2	ENSSSCG00000001422	peptidase S1 family	−1.01	<0.0001
C3	complement C3	ENSSSCG00000013551	—	−1.52	0.0006
C5	complement C5	ENSSSCG00000005512	—	−1.26	0.0010
NFKBIZ	NFKB inhibitor zeta	ENSSSCG00000011951	—	−1.19	<0.0001

### QRT-PCR verification results

**Figure 3:**
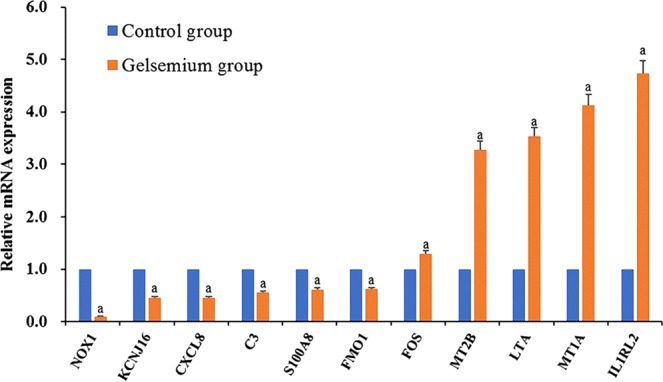
Relative expression of the candidate genes by qRT-PCR. Three samples were included in each group, and three replicates for each sample were performed. The relative expression level was measured by qRT-PCR. These data are expressed as the mean ± SD relative to the control. ^a^P < 0.05 compared with the control group.

The correlation analysis results of the qRT-PCR and RNA-seq data were similar (Fig. 3), which suggests that the transcriptome sequencing data are highly reproducible and accurate.

## Discussion

RNA-seq technology is an ideal high-throughput sequencing method that greatly improves the speed and efficiency of identifying new genes or biomarkers^12,13^. Therefore, in this study, the RNA-seq technique was used to determine the transcriptomic effects of *Gelsemium elegans* on the pig ileum, and 446 DEGs were obtained. A total of 237 genes were upregulated, and 209 genes were downregulated in the ileum tissue of the experimental group compared with that of the control group. At the same time, we found 14 important pathways. These results provide the basis for a better understanding of the molecular mechanisms of *Gelsemium elegans* in pigs.

The results of expression analysis of inflammatory response-related genes showed that the inflammatory genes in the experimental group were generally downregulated. First, genes associated with inflammatory-related receptors and signaling pathways, such as the cytokine–cytokine receptor interaction pathway and the IL-17 signaling pathway, suggesting that cytokine–cytokine receptor action is inhibited and that intercellular signal transmission is weakened under *Gelsemium* treatment^14,15^. Most of the genes enriched in inflammatory response-related pathways, such as the rheumatoid arthritis pathway, and related metabolic processes in inflammation, such as the complement and coagulation cascade pathways, were downregulated, indicating that the intestinal inflammatory response of the experimental group was inhibited.

The downregulated genes associated with the complement and coagulation cascades pathway in the experimental group compared with the control group were C4BPB, C4BPA, F11, C2, C3 and C5, among which C3 is a local inflammatory reaction process mediator that can induce smooth muscle contraction, increase vascular permeability and promote mast cells and leukocytes to release histamine. C3 also plays the role of an inflammatory cell chemical inducer in chronic inflammation. In addition, C5 is an effective chemokine that stimulates leukocyte movement and induces leukocytes to migrate to inflammatory sites. The complement and coagulation cascades were all downregulated, which may have been related to the inhibition of inflammatory reactions. This finding shows that *Gelsemium elegans* might reduce inflammatory cell infiltration by reducing the levels of cytokines related to the complement and coagulation cascades.

The IL-17 signaling pathway is involved in the pathogenesis of many inflammatory and autoimmune diseases, including rheumatoid arthritis^16^ and psoriasis. S100A8 is a calcium-zinc binding protein (with significantly downregulated expression) belonging to the low–molecular weight S100 protein family that participates in the migration process of neutrophils to inflammatory sites^1^, can bind with TLR4 and AGER receptors, and activates the MAP kinase and NF-K-B signaling processes, thus leading to expansion of the proinflammatory cascade and playing an important role in inflammatory process and immune response regulation.

In the rheumatoid arthritis pathway, a total of 5 DEGs were enriched, among which IL-8/CXCL-8 is a key mediator related to inflammation belonging to the CXC family of chemokines that is usually released in response to inflammatory stimulation. IL-8/CXCL-8 plays a key role in neutrophil recruitment, and degranulation^17^ is considered to be an inflammatory mediator of gingivitis^18^ and psoriasis. In a study of *Gelsemium elegans*, some researchers developed an injectable solution and oral liquid from a water extract of *Gelsemium elegans* and applied it to the treatment of psoriasis and neurodermatitis^19^. The downregulation of the CXCL-8 gene is consistent with the anti-inflammatory activities of *Gelsemium elegans* and emphasizes the role of CXCL-8 in the pharmacological activities of *Gelsemium elegans*. CSF2, also known as granulocyte-macrophage colony-stimulating factor (GM-CSF), is a monomeric glycoprotein. Clinical tests have revealed its proinflammatory effect. Studies have shown that CSF2 is highly expressed in joints with rheumatoid arthritis, and inflammation and injury can be reduced by blocking CSF2. IL-1α/IL1A, which are members of the IL-1 cytokine family together with IL-1β, can induce the release of IL-2 and mainly play a role in regulating and initiating inflammatory reactions. Studies have found that blocking the activity of IL-1α is likely to be useful for the treatment of skin diseases such as acne^20^. IL-1β is an important mediator of inflammatory reactions that can induce many kinds of self-inflammatory diseases. Experiments have found that intestinal ecological imbalance can induce osteomyelitis in an IL-1β-dependent manner^21^.

A large number of studies have indicated that *Gelsemium elegans* has strong anti-inflammatory activity^22–24^. Researchers have used transcriptome analysis to study SH-SY5Y neurons in humans treated with low concentrations of *Gelsemium* alkaloids and found significant changes in related regulatory genes of the inflammatory response signaling pathway^23^. Some researchers have also found that medium and high doses of *Gelsemium* could significantly inhibit the mitosis process in vaginal epithelial cells and promote the formation of epidermal granules in mouse tail scales. At the same time, the level of IL-2 in the serum of experimental mice was found to be significantly reduced in three dose groups^24^. Koumine, which is one of the major *Gelsemium* alkaloids, has been found to reduce the levels of the proinflammatory factors TNF and IL-1, attenuate collagen-induced increases in fibrillary protein content, and inhibit astrocyte activation and proinflammatory factor–mediated pain^25,26^.

Using qPCR and Western blot analysis, Yuan *et al*. found that koumine can inhibit the release of inflammatory mediators and cytokines such as TNF-α, IL-6 and IL-1β^27^. Similarly, studies have shown that IL-1α^28^, CXCL8^29^, CSF2^30^, C3^31^, C5^32^ and S100A8^33^ were consistent at the mRNA and protein levels. However, Yang *et al*. found that koumine can selectively inhibit the proliferation of T cells, especially CD4+ T (Th) cells, reducing the levels of inflammatory cytokines (Th1 cytokines IFN-γ, IL-1β, 2, 6, 12, TNF-α and Th17-α cytokines IL-17A)^34^. Aggarwal A. *et al*. found that rheumatoid arthritis is closely related to polarity deviation in Th cells and systemic disorder of inflammatory and anti-inflammatory cell networks^35^. It is worth noting that macrophages can release IL-1α, which acts on Th cells to regulate their activity. These studies suggest that *Gelsemium elegans* may regulate the polarity deviation of Th cells by inhibiting the expression of IL-1α, thus reducing the production of inflammatory factors. In this study, the expression of many inflammatory cytokine genes was inhibited, and the levels of these cytokines entering the blood to reach corresponding lesions were decreased; these changes achieved an anti-inflammatory effect by restoring the balance of the network.

## Conclusions

In this study, we reported the ileum transcriptome profiles of three-way hybrid weaned piglets fed two different diets (a complete diet without antibacterial drugs and a complete diet containing 2% *Gelsemium elegans* herb without antibacterial drugs) and carried out detection and DEG identification. The results showed that *Gelsemium elegans* affected many inflammatory and immune pathways, including biological processes such as defense responses, inflammation and immune responses. More importantly, this study identified several important genes (e.g., CXCL-8, IL1A, and CSF2). These data provide a clearer understanding for elucidating the molecular mechanisms of the pharmacological actions of *Gelsemium*, such as its anti-inflammatory effects.

## Materials and Methods

### Animals and treatments

In this study, 6 healthy castrated male ternary hybrid piglets approximately 60 days old and weighing 20 ± 2 kg were obtained from Hunan Xinwufeng, Yong’an branch (Hunan, China), and randomly divided into control and *Gelsemium elegans* treatment groups. The control group was a blank control group fed a complete diet without antibacterial drugs, and the *Gelsemium elegans* group was an experimental group. The diet was prepared by adding 2% *Gelsemium elegans* powder to a complete diet without antibacterial drugs. The formula and nutritional level of the complete diet are shown in Table 4. After 45 days of continuous feeding, administration was stopped, and all piglets were fasted for 24 hours. The piglets were slaughtered within one day after stopping the drug, and a sterile blade was used to take ileum tissue from the control group and the experimental group. The tissue samples were washed in distilled water to remove blood and placed into centrifuge tubes. The tubes were sealed with sealing film and quickly placed in liquid nitrogen for storage. The samples were stored at −80 °C until RNA extraction. This experiment was carried out in accordance with the Guidelines for the Care and Use of Laboratory Animals of China and approved by the Animal Care and Use Committee of the Institute of Subtropical Agriculture, Chinese Academy of Sciences (IACUC# 201302).

**Table 4:** Composition and nutritional level of the complete diet.

Ingredients	Ratio (%)
Corn	52.99
Expanded corn	10.00
Soybean meal	20.00
Fish meal	1.50
Soybean oil	0.80
Fine powder	0.72
Salt	0.30
Sucrose	2.50
Premix^a^	2.59
Total	100.00
**Nutrient levels** ^**b**^	
DE (MJ/kg)	14.20
Crude Protein(%)	19.00
Calcium (%)	0.80
Available phosphorus (%)	0.50
Lysine (%)	1.40
Methionine (%)	0.84
L-Threonine (%)	0.40

^(a)^Premix is provided per kilogram of daily ration: VA 2200 IU; VD 220 IU; VE 62 mg; VK3 3.00 mg; VB1 3.00 mg; VB6 3.60 mg; VB12 0.02 mg; Biotin 0.45 mg; Pantothenic acid 10 mg; Nicotinic acid 30 mg; Choline chloride 500 mg; Mn 50 mg; Fe 150 mg; Zn 2200 mg; Cu 200 mg; I 0.3 mg; Se 0.3 mg. ^(b)^The crude protein level in the table is the measured value, and the other is the calculated value.

### Extraction of total RNA

Total RNA was isolated from each sample using TRIzol reagent (Invitrogen, Carlsbad, CA, USA) according to the manufacturer’s protocol. The integrity and purity of the RNA were detected by agarose gel electrophoresis (Tianneng, China) and with a NanoDrop (Thermo, USA), and the RNA concentration was accurately quantified with a Qubit instrument (Invitrogen, USA).

### Construction of the sequencing library

The mRNA was enriched by magnetic beads with Oligo(dT), and then, the mRNA was randomly broken into short fragments of approximately 200 bp. Using mRNA as a template, first-strand cDNA was synthesized by reverse transcription using random primers. For synthesis of the second strand of cDNA, dTTP in the dNTP mixture was replaced by dUTP. The cDNA product was purified with AMPure XP beads (Beckman, USA); then, the double-stranded ends were blunted with End Repair Mix, and poly-A tails and sequencing linkers were added. The USER enzyme was used to digest the double-stranded cDNA to make a library containing only single-stranded cDNA. PCR (Life, USA) enrichment was performed, and the final cDNA library was obtained after 15 cycles of amplification.

### Transcriptome sequencing

An Illumina HiSeq sequencing platform (Illumina, USA) was used, and the 2*150 sequencing mode was adopted to construct a qualified cDNA library for double-terminal (PE) sequencing by Shanghai Jingneng Biotechnology Limited (Shanghai, China).

### Processing and analysis of sequencing results

According to the distribution feature that the low-mass fraction of Illumina sequencing data is usually concentrated at the end, the raw sequencing data were filtered to obtain clean reads. Trim Galore (http://www.bioinformatics.babraham.ac.uk/projects/trim_galore/) software was used to remove the linker sequence fragments and low-mass fragments from the 3’ ends of the raw data. Finally, high-quality clean reads were obtained, and the clean reads and pig reference genome sequences were sequence-matched with STAR software^36^. The obtained alignment results were based on the positional information files of the known transcripts on the genome, and transcripts with expression values of 0 were removed with StringTie software^37^. Then, the transcripts for all samples assembled with StringTie were reassembled to obtain new transcripts. BLASTX software was used to carry out sequence alignment with the UniProt (http://www.uniprot.org/downloads), GO (http://geneontology.org/) and KEGG (http://www.genome.jp/) databases to obtain corresponding unigene functional annotation information.

### Differential expression analysis

The FPKM values were used to calculate the gene expression levels in the samples, and differential expression was screened between the experimental group samples and the control group samples using DESeq. 2 software. DEGs were identified by using a log2|fold change| ≥ 1, a P-value ≤ 0.05 and an FDR (adjusted for P-value) ≤ 0.05 as the screening conditions. GO functional enrichment analysis^38^ and KEGG pathway enrichment analysis^39^ for the DEGs were performed.

### Validation of RNA-seq data by quantitative real-time PCR (qRT-PCR)

Validation was performed by using the same RNA used for sequencing. qRT-PCR was performed on selected DEGs, including C3, CXCL-8, FOS, LTA, S100A8, IL1RL2, NOX1, KCNJ16, FMO1, MT2B and MT1A. Primer sequences were designed using the NCBI primer design tool and synthesized by Qingke Xinye Biotechnology Limited (Changsha, China). Fluorescence quantitative detection was performed using a TIANLONG TL988 fluorescence quantitative PCR instrument (Xi’an Tianlong Technology Limited). Each sample was analyzed in triplicate, and the expression of the target genes was standardized by GAPDH expression. The reaction protocol comprised one cycle of 95 °C for 1 min followed by forty cycles of 95 °C for 10 s, 60 °C for 5 s and 72 °C for 10 s. The gene expression was calculated by using the comparative Ct (2^−ΔΔCt^) method.

## Data Availability

The datasets analyzed during the current study are available in the NCBI Sequence Read Archive (SRA) under BioProject ID: PRJNA 552943.
